# Vaccine Effectiveness Estimates Against Influenza A(H3N2)-Associated Hospitalized Severe Acute Respiratory Infections in Beijing, China, 2025/26 Influenza Season

**DOI:** 10.3390/vaccines14050457

**Published:** 2026-05-20

**Authors:** Chunna Ma, Jiaojiao Zhang, Jiaxin Ma, Wei Duan, Yingying Wang, Xiaodi Hu, Jia Li, Lu Zhang, Yuanzhi Di, Shuning Yan, Peng Yang, Quanyi Wang, Ying Shen, Daitao Zhang

**Affiliations:** 1 Beijing Research Center for Respiratory Infectious Diseases, Beijing 100013, China; ma_chun_na@126.com (C.M.);; 2 Beijing Key Laboratory of Surveillance, Early Warning and Pathogen Research on Emerging Infectious Diseases, Beijing Center for Disease Prevention and Control, Beijing 100013, China; 3 Institute for Infectious Disease and Endemic Disease Control, Beijing Center for Disease Prevention and Control, Beijing 100013, China; 4 School of Public Health, Capital Medical University, Beijing 100069, China

**Keywords:** influenza, vaccine effectiveness, hospitalization, test-negative design, China

## Abstract

Background: Data on influenza vaccine effectiveness (VE) against hospitalized severe acute respiratory infection (SARI), particularly in Asia, remain limited for the 2025/26 Northern Hemisphere influenza season. This study aimed to evaluate real-world VE against A(H3N2)-associated SARI hospitalization and provide timely, locally relevant evidence to inform seasonal influenza vaccination policy. Methods: A test-negative design was used to estimate VE against influenza A(H3N2)-associated SARI hospitalization in Beijing, China, from 10 November 2025 to 18 January 2026. VE was estimated by comparing the odds of influenza vaccination between case-patients (those who tested positive for A(H3N2)) with controls (those who tested negative for influenza). Results: Among 1883 enrolled SARI inpatients, 220 (11.7%) tested positive for influenza A(H3N2). Overall vaccination coverage was 11.4%, with the highest coverage observed among children aged 5–17 years (29.6%). Influenza positivity was higher among rural residents, patients with pneumonia or hypoxemia, and those with symptom onset in November. The adjusted overall VE was 7.5% (95% CI: −45.8% to 43.3%). Moderate VE was observed among children aged 5–17 years (45.4%, 95% CI: −33.6% to 79.5%), although the confidence interval included zero and the estimate was not statistically significant. Negative VE estimates were observed among younger children and older adults. Among patients with underlying respiratory conditions, VE was 75.4% (95% CI: −27.4% to 98.7%), although this estimate was also not statistically significant. Conclusions: During the 2025/26 influenza season in Beijing, VE against A(H3N2)-associated SARI hospitalization was suboptimal. Moderate protection was observed among children aged 5–17 years, the group with the highest vaccination coverage, but the estimate was not statistically significant. The low overall VE may be attributable to antigenic mismatch between vaccine and circulating strains, as well as low population-level vaccination coverage. These findings highlight the need to improve vaccine formulations and increase vaccination coverage, particularly among adults and older populations.

## 1. Introduction

During the 2025/26 influenza season, global influenza activity showed regional variation in epidemic timing, circulating subtypes, and subclades. In the United States, influenza activity started later but was more intense than in the previous three seasons, peaking in Week 52 of 2025. Influenza A(H3N2) was the dominant subtype, accounting for 85.4% of detections by Week 8 of 2026, and 92.6% of A(H3N2) viruses belonged to subclade K [1]. The proportion of B/Victoria lineage viruses began to increase from Week 3 of 2026 onward [1]. In Europe, influenza activity began 3–4 weeks earlier than in the previous two seasons and was associated with higher test positivity [2]. A(H3N2) was the dominant strain, accounting for approximately 73.0% of detections by Week 9 of 2026 [3], and 90% of these viruses belonged to subclade K [3], which showed substantial antigenic mismatch with the Northern Hemisphere-recommended vaccine strains [4]. As of Week 8 of 2026, A(H3N2) remained the dominant subtype, followed by A(H1N1)pdm09. In mainland China, the influenza peak occurred in Week 49 of 2025, earlier than in the previous three seasons. A(H3N2) was overwhelmingly dominant before Week 52 of 2025, with only 23.2% of viruses antigenically similar to the Northern Hemisphere-recommended egg-adapted vaccine strain (determined by hemagglutination inhibition assay) [5]. Subsequently, the proportion of B/Victoria lineage viruses gradually increased from Week 1 of 2026 and exceeded 50% by Week 9 of 2026 [5].

Influenza circulates periodically and spreads rapidly worldwide. Severe acute respiratory infection (SARI) caused by influenza is an important clinical outcome leading to hospitalization and death, and it continues to impose a substantial global public health burden. Previous studies have shown that children aged <5 years [6], adults aged ≥65 years [7,8], and individuals with underlying conditions such as chronic obstructive pulmonary disease (COPD) [9] have higher rates of influenza-associated SARI hospitalization and severe outcomes than the general population. These findings underscore the need for effective interventions in high-risk groups. Influenza vaccination reduces the incidence of laboratory-confirmed influenza and lowers the risk of influenza-related severe complications and death, although vaccine effectiveness (VE) varies. Vaccination has also been shown to reduce the risk of hospitalization in specific high-risk populations [10,11]. However, protection differs by influenza virus subtype, and heterogeneous VE estimates have been reported across geographic regions and healthcare settings.

Timely evidence on VE against hospitalized SARI in Asia remains limited, which may hinder the dynamic and precise adjustment of influenza prevention and control strategies. Therefore, this study aimed to estimate influenza VE against hospitalized SARI during the 2025/26 Northern Hemisphere influenza season. By analyzing laboratory-confirmed hospitalized SARI cases, we estimated overall VE, and examined variation in effectiveness by age group and underlying medical conditions. We also explored the potential influence of age, comorbidities, and other factors on VE. The findings provide evidence to support the refinement of prevention and control measures during an influenza season dominated by A(H3N2), and may inform vaccination strategies, interventions for high-risk populations, and public health resource allocation in future influenza seasons.

## 2. Methods

### 2.1. Study Design, Participants, and Laboratory Testing

A test-negative design was used to assess influenza VE against influenza-associated SARI hospitalization in Beijing, China. SARI cases were collected from sentinel hospitals participating in the Multi-Pathogen Surveillance System, which includes 19 sentinel hospitals and 16 network laboratories in Beijing. Within this system, trained healthcare professionals randomly collected a total of 10 respiratory tract specimens per week from eligible SARI patients in designated hospital wards, including respiratory, pediatric internal medicine, critical care, infection, and emergency ICU at each sentinel hospital. Specimen types consisted of deep sputum, bronchoalveolar lavage fluid, oropharyngeal swabs, nasal swabs, and nasopharyngeal swabs. Specimens were transported to designated network laboratories at 4 °C. Influenza virus was detected by real-time RT-PCR following the standard procedures recommended by the WHO Collaborating Center for Reference and Research on Influenza at the Chinese National Influenza Center (CNIC) [12]. All specimens were subjected to real-time RT-PCR to screen influenza A and B viruses. Positive specimens were further subtyped into A(H1N1)pdm09, A(H3N2), as well as Victoria and Yamagata lineages of influenza B. The positivity cutoff was defined as specified in the manufacturer’s instructions.

The influenza epidemic period was defined as consecutive weeks during which the weekly influenza positivity rate reached or exceeded 40% of the seasonal peak value [13]. The “peak week” was defined as the week with the highest influenza positivity rate in Beijing’s virological surveillance data during the 2025/26 influenza season. Based on this definition, the epidemic period in this study extended from Week 46 (10 November–16 November 2025) to Week 3 (12 January–18 January 2026).

**Figure 1:**
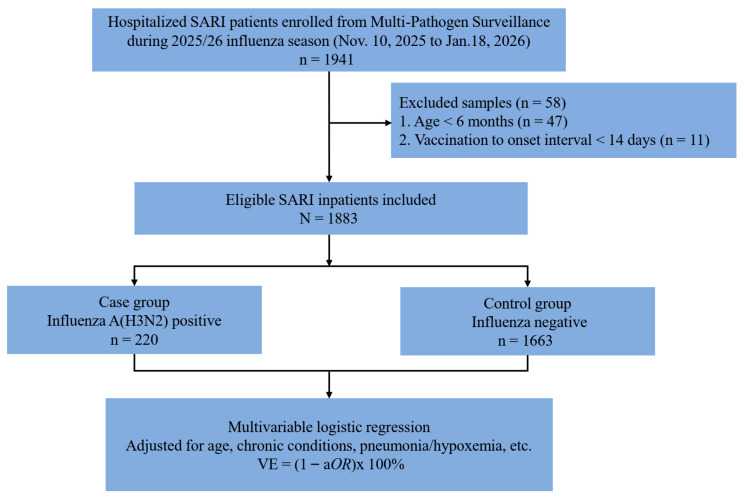
Flowchart of the test-negative design for estimating influenza vaccine effectiveness (VE) against influenza A(H3N2)-associated hospitalized SARI in Beijing, China, during the 2025/26 influenza season. Under this design, hospitalized SARI patients were enrolled and tested for influenza by real-time RT-PCR. Participants were classified into case (A(H3N2)-positive) or control (influenza-negative) groups. VE was calculated as (1 − adjusted odds ratio) × 100% using multivariable logistic regression.

Hospitalized SARI cases were defined as patients who, at admission or within 48 h of hospitalization, presented with acute onset of illness, fever during the current illness (temperature ≥ 38.0 °C), cough, and illness duration ≤ 10 days. Although SARI can be caused by a range of respiratory pathogens, this study focused on influenza-associated SARI hospitalization. Multi-pathogen surveillance data showed that influenza viruses were the predominant respiratory pathogens circulating during the study period. Only participants aged ≥6 months were included, as influenza vaccines in China are approved only for this age group. Participants were excluded if vaccination occurred after symptom onset, or within 14 days before symptom onset (Figure 1).

### 2.2. Data Collection

Using a standardized online system, healthcare professionals at sentinel hospitals systematically collected epidemiological data from sampled patients. These data included demographic characteristics (age and sex), underlying medical conditions, and clinical information (visiting date, sampling date, presence of pneumonia/hypoxemia (defined as resting SpO_2_ ≤ 90% on room air), and antiviral drugs use). Underlying medical conditions included respiratory diseases (asthma, chronic tracheitis/bronchitis, and chronic obstructive pulmonary disease); cardiovascular diseases (hypertension and coronary heart disease); cerebrovascular diseases (cerebrovascular accident or stroke); metabolic disorders (diabetes and hyperlipidemia); as well as HIV/AIDS, malignancies, and, renal, hepatic, neurological, and immune system diseases. Vaccination status was obtained from the Beijing Immunization Information Management System.

### 2.3. Statistical Analysis

Statistical analyses were performed using R software (version 4.4.1). Frequencies and percentages were used to summarize demographic and clinical characteristics by case–control status and vaccination status. Statistical significance was defined as a two-sided *p*-value < 0.05.

Based on the test-negative design, multivariable logistic regression was used to estimate adjusted odds ratios (aORs) for vaccination status among influenza A(H3N2)-positive cases compared with influenza-negative controls. Models were adjusted for potential confounding factors that were imbalanced between the two groups. Univariable analyses were first performed to identify candidate confounders, using Fisher’s exact test for categorical variables. Variables with *p* < 0.1 in univariable analyses were considered for inclusion in the multivariable models. VE was calculated as (1 − a*OR*) × 100%. Subgroup analyses of VE against influenza A(H3N2)-associated SARI hospitalization were further stratified by age group (0.5–4, 5–17, 18–59, and ≥60 years), presence of chronic conditions, respiratory conditions and pneumonia/hypoxemia.

## 3. Results

### 3.1. Influenza Activity and Study Population

Influenza-positive cases among hospitalized SARI patients were first identified in Week 37 of 2025 (8 September–14 September). The positivity increased steadily from Week 42 (13 October–19 October 2025), peaked at 24.2% in Week 47 (17 November–23 November 2025), and declined sharply thereafter.

According to the predefined epidemic period definition, a total of 1941 hospitalized SARI patients were enrolled from sentinel hospitals participating in the Multi-Pathogen Surveillance System between Week 46 (10 November–16 November 2025) and Week 3 (12 January–18 January 2026). Among these patients, 47 were under 6 months of age, and 11 had symptom onset before vaccination or within 14 days after vaccination. After these exclusions, 1883 participants were included in the study. Among the patients, only A(H3N2) was detected; no other subtypes (e.g., A(H1N1)) or influenza B lineages were identified. Of these, 220 patients (11.7%) tested positive for influenza A(H3N2) and were classified as cases, while 1663 patients (88.3%) tested negative for influenza and were classified as controls (Figure 1).

### 3.2. Participant Characteristics, Influenza Positivity, and Vaccination Coverage

Baseline characteristics and positivity rates of study participants, stratified by different variables, are presented in Table 1. The overall influenza positivity rate (all confirmed cases were influenza A(H3N2)) was 11.7%. Influenza positivity was slightly higher among vaccinated individuals than among unvaccinated individuals, although the difference was not statistically significant (13.5% vs. 11.5%; *p* = 0.368). Positivity for influenza A(H3N2) was significantly higher among rural residents than among urban residents (13.7% vs. 8.9%; *p* = 0.001). No significant difference was observed between males and females (12.0% vs. 11.2%; *p* = 0.610). The median age was 67.0 years (interquartile range, IQR: 67.0) in the influenza-positive group and 68.0 years (IQR: 50.5) in the influenza-negative group.

When stratified by age group, the highest positivity rate was observed among children aged 5–17 years (16.6%), followed by adults aged ≥60 years (11.4%), children aged 0.5–4 years (11.3%), and adults aged 18–59 years (9.5%), although the difference was not statistically significant (*p* = 0.127). No significant differences in positivity were observed between individuals with and without any chronic condition (11.1% vs. 12.0%; *p* = 0.552), between those with and without respiratory conditions (9.0% vs. 12.1%; *p* = 0.159), or between those with and without hypertension, cardiovascular disease, or cerebrovascular disease (10.9% vs. 11.9%; *p* = 0.591). Positivity was higher among patients with pneumonia/hypoxemia than among those without these features (13.6% vs. 8.3%; *p* < 0.001). By calendar month of SARI symptom onset, the highest positivity rate was observed in November 2025 (18.0%), followed by December 2025 (10.9%), and January 2026 (4.8%; *p* < 0.001). No significant differences were observed by interval from SARI onset to sampling (0–3 days: 13.3%, 4–7 days: 10.4%, ≥8 days: 11.4%; *p* = 0.272) or by antiviral drugs use before sampling (17.9% vs. 11.4%; *p* = 0.363).

Baseline vaccination coverage, stratified by demographic and clinical factors, is summarized in Table 1. Overall influenza vaccination coverage was 11.4%. All vaccines used in this study were egg-based inactivated influenza vaccines, of which 97.2% were split-virus vaccines and 2.8% were subunit vaccines (purified HA). Vaccination coverage was significantly higher among rural residents than urban residents (13.8% vs. 8.1%; *p* < 0.001). By age group, the highest vaccination coverage was observed among children aged 5–17 years (29.6%), followed by adults aged ≥ 60 years (11.8%), children aged 0.5–4 years (5.3%), and adults aged 18–59 years (1.5%; *p* < 0.001). No significant differences in vaccination coverage were observed between males and females (11.9% vs. 10.7%; *p* = 0.392), individuals with and without chronic conditions (10.6% vs. 11.8%; *p* = 0.425), individuals with and without respiratory conditions (12.8% vs. 10.9%; *p* = 0.359), individuals with and without hypertension, cardiovascular disease, or cerebrovascular disease (10.3% vs. 11.7%; *p* = 0.372), those with and without pneumonia/hypoxemia (12.5% vs. 9.6%; *p* = 0.059), or those who did and did not receive anti-influenza antiviral drugs before sampling (0% vs. 11.4%; *p* = 0.065).

### 3.3. Influenza Vaccine Effectiveness

Among hospitalized patients with SARI, 13.5% of vaccinated individuals tested positive for influenza A(H3N2), compared with 11.5% of unvaccinated individuals (Table 1). The unadjusted VE against influenza A(H3N2)-associated SARI hospitalization was −20.6% (95% CI: −80.7% to 22.1%). After adjustment for potential confounders, including region, calendar month of symptom onset, age group, pneumonia or hypoxemia, and antiviral drugs use before sampling, the adjusted VE was 7.5% (95% CI: −45.8% to 43.3%) (Table 2).

Subgroup analyses of VE against influenza A(H3N2) are presented in Table 2. The adjusted VE among children aged 5–17 years was 45.4% (95% CI: −33.6% to 79.5%). Among adults aged ≥60 years, the adjusted VE was −16.1% (95% CI: −95.3% to 34.4%). Among children aged 0.5–4 years, the adjusted VE was −102.5% (95% CI: −626.6% to 57.1%). VE could not be estimated for adults aged 18–59 years because no influenza-positive cases were observed among vaccinated individuals in this age group.

Among participants with any chronic condition, the adjusted VE was −44.8% (95% CI: −163.6% to 24.7%). Among those without chronic conditions, the adjusted VE suggested a protective effect, at 41.3% (95% CI: −16.0% to 72.6%). Among participants with respiratory conditions, the adjusted VE was 75.4% (95% CI: −27.4% to 98.7%), whereas among those without respiratory conditions, the adjusted VE was 1.4% (95% CI: −60.7% to 41.7%). The adjusted VE was 0.2% (95% CI: −68.1% to 43.0%) among participants with pneumonia or hypoxemia and 25.1% (95% CI: −85.6% to 75.4%) among those without these features (Table 2).

## 4. Discussion

This study evaluated influenza VE against A(H3N2)-associated SARI hospitalization in Beijing during the 2025/26 season. The adjusted overall VE was 7.5% (95% CI: −45.8% to 43.3%), indicating suboptimal protection. This finding is consistent with a previous Beijing-based study of hospitalized patients during the 2017/18 season, which reported poor VE against A(H3N2) (−37.7%), compared with VE against A(H1N1)pdm09 (29.2%) [14]. In contrast, our results differ from another interim outpatient study conducted in Beijing during the same season, which reported modest VE against A(H3N2) of 23.5% [15]. Our findings also differ substantially from contemporaneous estimates from the European Union (VE: 52%, 95% CI: 29–69% against A(H3N2) among ARI/ILI) [2], England (VE against A(H3N2)-associated hospitalization: 72.8% in children/adolescents, 66.3% in adults aged 18–64 years, and 31.7% in adults ≥65 years) [16] and France (interim VE against co-circulation of A(H3N2) and A(H1N1)pdm09 among outpatients was 36.4%) [17]. These discrepancies may be explained by two main factors.

First, differences in vaccine platforms and immunization strategies may have contributed to the observed heterogeneity. In China, seasonal influenza vaccination is primarily based on egg-derived trivalent and quadrivalent inactivated vaccines, including split-virus and subunit formulations, while trivalent live attenuated influenza vaccines (LAIVs) are available as a supplementary option [18]. In contrast, England prioritizes enhanced vaccines for older adults, including adjuvanted inactivated influenza vaccine (aIIV), high-dose inactivated influenza vaccine (IIV-HD), and recombinant influenza vaccine (IIVr); and prefers LAIV or cell culture-based inactivated influenza vaccine (IIVc) for children and adolescents [19]. Across the EU/EEA, 27 countries fully fund vaccination for older adults, and 12 specifically use enhanced vaccine products, such as adjuvanted, high-dose, or cell culture-based vaccines, for this group [20]. A(H3N2) viruses are prone to adaptive mutations during egg-based cultivation, which may lead to antigenic mismatch [21,22]. Studies from England have shown that, for circulating A (H3N2) subclade K viruses, hemagglutination inhibition titers against egg-cultured strains (e.g., A/Croatia/10136RV/2023) decreased by >32-fold, compared with a ≥8-fold for cell-cultured strains (e.g., A/District of Columbia/27/2023), suggesting poorer antigenic matching for egg-based strains [16].

Consistently, data from the Chinese National Influenza Center indicated that 39.5% of circulating A(H3N2) viruses were egg-culture-like, compared with 72.5% that were cell-culture-like [5]. Expert estimates suggest that avoiding egg-adaptive changes could increase VE against A(H3N2) by up to 16% among individuals aged <65 years [22]. Previous studies have also shown that IIV-HD, aIIV, and IIVr elicit stronger antibody responses and provide better protection against laboratory-confirmed influenza or influenza-related hospitalization than conventional egg-based vaccines [23,24,25,26].

Second, low population-level vaccination coverage may have affected the precision and stability of our VE estimates. In this study, vaccination coverage was 5.3% among children aged 0.5–4 years, 29.6% among those aged 5–17 years, 1.5% among adults aged 18–59 years, and 11.8% among adults aged ≥60 years. The particularly low coverage among young and middle-aged adults resulted in few vaccinated cases and wide confidence intervals. In contrast, the England study reported vaccination coverage of 34% among children aged 2–3 years, 62% among adults aged ≥65 years, and 29% among clinically at-risk individuals aged 6 months to <65 years, providing a more robust foundation for herd immunity [16].

Despite cross-regional differences in absolute VE estimates, all studies broadly showed higher VE in children/adolescents and lower VE in older adults. Our study (hospitalized SARI) found VE of −102.5% (0.5–4 years), 45.4% (5–17 years), and −16.1% (≥60 years); the England study (hospitalized) reported 72.8% (2–17 years), 66.3% (18–64 years), and 31.7% (≥65 years) [16]; the France study (outpatient) reported 57.2% (0–17 years), 45.1% (18–64 years), and 27.7% (≥65 years) [17]; the EU study (AIRI/ILI) reported 52% (0–17 years) and 57% (18–64 years) [2]. Overall, most studies showed a similar age-related pattern, with the highest VE among children and adolescents, moderate protection among adults, and the lowest VE among older adults. This finding has two public health implications. First, children and adolescents, who have high transmission potential and generally robust vaccine-induced immune responses, should remain a core target group for influenza vaccination. Second, older adults, who accounted for 62.3% of hospitalized SARI cases in our study and may have weaker immune responses to conventional influenza vaccines, urgently require improved or enhanced vaccine formulations.

Among patients with chronic respiratory diseases, the adjusted VE was 75.4% (3 positive cases among 20 vaccinated individuals). Although the confidence interval was wide because of the small sample size, the point estimate suggests potential protection, consistent with previous studies showing that influenza vaccination can reduce the risk of severe COPD exacerbations and influenza-related hospitalization among high-risk populations [27,28]. Conversely, VE was −44.8% among individuals with any chronic condition and 41.3% among those without chronic condition, suggesting stronger protection among healthier individuals. However, these subgroup findings should be interpreted cautiously because of limited sample sizes and imprecise estimates.

This study has some limitations. First, small sample sizes in several subgroups, including patients with chronic respiratory diseases and adults aged 18–59 years, resulted in wide confidence intervals and limited statistical precision. Second, the short post-vaccination observation period precluded assessment of potential VE waning over time.

## 5. Conclusions

In conclusion, VE against A(H3N2)-associated SARI hospitalization in Beijing, during the 2025/26 season was suboptimal. This may be mainly attributable to antigenic mismatch related to egg-based vaccine platforms and potentially low population-level vaccination coverage. To improve influenza vaccine protection in China, it is important to optimize vaccine platforms, facilitate the authorization and use of enhanced vaccines, develop targeted vaccination strategies for different age groups and subpopulations, and strengthen public education to increase vaccination coverage, particularly among young and middle-aged adults and older populations.

## Figures and Tables

**Table 1:** Characteristics of hospitalized patients with SARI by influenza test status and vaccination status during the 2025/26 season in Beijing, China.

Characteristic	Total, No. (Col. %) N = 1883	Influenza A(H3N2) Test Result			Influenza Vaccination Status		
		Positive, No. (Row %) N = 220	Negative, No. (Row %) N = 1663	*p*-Value	Vaccinated, No. (Row %) N = 215	Unvaccinated, No. (Row %) N = 1668	*p*-Value
Vaccination in 2025–26 season, n (%)				0.368			
Yes	215 (11.4)	29 (13.5)	186 (86.5)		215 (100)	0	
No	1668 (88.6)	191 (11.5)	1477 (88.5)		0	1668 (100)	
Site, n (%)				0.001			<0.001
Urban	788 (41.8)	70 (8.9)	718 (91.1)		64 (8.1)	724 (91.9)	
Rural	1095 (58.2)	150 (13.7)	945 (86.3)		151 (13.8)	944 (86.2)	
Sex, n (%)				0.610			0.392
Male	1114 (59.2)	134 (12.0)	980 (88.0)		133 (11.9)	981 (88.1)	
Female	769 (40.8)	86 (11.2)	683 (88.8)		82 (10.7)	687 (89.3)	
Age (years), Median (IQR)	68.0 (53.0)	67.0 (67.0)	68.0 (50.5)	0.357	70.0 (68.0)	67.0 (48.3)	0.431
Age group, n (%)				0.127			<0.001
0.5–4 years	247 (13.1)	28 (11.3)	219 (88.7)		13 (5.3)	234 (94.7)	
5–17 years	199 (10.6)	33 (16.6)	166 (83.4)		59 (29.6)	140 (70.4)	
18–59 years	264 (14.0)	25 (9.5)	239 (90.5)		4 (1.5)	260 (98.5)	
≥60 years	1173 (62.3)	134 (11.4)	1039 (88.6)		139 (11.8)	1034 (88.2)	
Any chronic condition, n (%)				0.552			0.425
Any	895 (50.7)	99 (11.1)	796 (88.9)		95 (10.6)	800 (89.4)	
None	872 (49.3)	105 (12.0)	767 (88.0)		103 (11.8)	769 (88.2)	
Respiratory condition, n (%)				0.159			0.359
Any	290 (16.4)	26 (9.0)	264 (91.0)		37 (12.8)	253 (87.2)	
None	1477 (83.6)	178 (12.1)	1299 (87.9)		161 (10.9)	1316 (89.1)	
Hypertension, cardiovascular disease or cerebrovascular disease, n (%)				0.591			0.372
Any	667 (37.7)	73 (10.9)	594 (89.1)		69 (10.3)	598 (89.7)	
None	1100 (62.3)	131 (11.9)	969 (88.1)		129 (11.7)	971 (88.3)	
Pneumonia/hypoxemia, n (%)				<0.001			0.059
Yes	1195 (63.5)	163 (13.6)	1032 (86.4)		149 (12.5)	1046 (87.5)	
No	688 (36.5)	57 (8.3)	631 (91.7)		66 (9.6)	622 (90.4)	
Month of SARI symptom onset, n (%)				<0.001			0.330
October 2025	600 (31.9)	108 (18.0)	492 (82.0)		69 (11.5)	531 (88.5)	
November 2025	825 (43.8)	90 (10.9)	735 (89.1)		102 (12.4)	723 (87.6)	
December 2025	458 (24.3)	22 (4.8)	436 (95.2)		44 (9.6)	414 (90.4)	
Time interval between SARI onset and sampling, n (%)				0.272			0.409
0–3 days	649 (34.5)	86 (13.3)	563 (86.7)		66 (10.2)	583 (89.8)	
4–7 days	653 (34.7)	68 (10.4)	585 (89.6)		76 (11.6)	577 (88.4)	
≥8 days	581 (30.9)	66 (11.4)	515 (88.6)		73 (12.6)	508 (87.4)	
Antiviral drugs use before sampling				0.363			0.065
Yes	28 (1.6)	5 (17.9)	23 (82.1)		0 (0.0)	28 (100.0)	
No	1739 (98.4)	199 (11.4)	1540 (88.6)		198 (11.4)	1541 (88.6)	

Respiratory conditions include asthma, chronic tracheitis/bronchitis, and chronic obstructive pulmonary disease (COPD). Hypoxemia was defined as resting SpO_2_ ≤ 90% on room air.

**Table 2:** Estimated vaccine effectiveness against influenza A(H3N2)-associated hospitalized SARI in Beijing, China, during the 2025/26 influenza season.

Groups	A(H3N2) Positives Vaccinated No./Total (%)	Influenza Negatives Vaccinated No./Total (%)	Adjusted VE% (95% CI)
Overall	25/183 (13.7)	129/1131 (11.4)	7.5 (−45.8, 43.3)
Age group			
0.5–4 years	3/25 (12.0)	8/145 (5.5)	−102.5 (−626.6, 57.1)
5–17 years	8/32 (25.0)	37/100 (37.0)	45.4 (−33.6, 79.5)
18–59 years	0/18 (0.0)	4/184 (2.2)	
≥60 years	14/108 (13.0)	80/702 (11.4)	−16.1 (−95.3, 34.4)
Any chronic conditions			
Any	13/81 (16.0)	56/543 (10.3)	−44.8 (−163.6, 24.7)
None	11/91 (12.1)	64/533 (12.0)	41.3 (−16.0, 72.6)
Respiratory conditions			
Any	3/20 (15.0)	25/177 (14.1)	75.4 (−27.4, 98.7)
None	21/152 (13.8)	95/899 (10.6)	1.4 (−60.7, 41.7)
Pneumonia/hypoxemia			
Yes	20/133 (15.0)	85/666 (12.8)	0.2 (−68.1, 43.0)
No	5/50 (10.0)	44/465 (9.5)	25.1 (−85.6, 75.4)

## Data Availability

The original database containing confidential patient information cannot be made publicly available. The anonymized data used in this study are available upon reasonable request to the corresponding authors.
